# Factorial Validity and Invariance of the 7-Item Generalized Anxiety Disorder Scale (GAD-7) Among Populations With and Without Self-Reported Psychiatric Diagnostic Status

**DOI:** 10.3389/fpsyg.2018.01741

**Published:** 2018-09-19

**Authors:** Satomi Doi, Masaya Ito, Yoshitake Takebayashi, Kumiko Muramatsu, Masaru Horikoshi

**Affiliations:** ^1^Department of Global Health Promotion, Tokyo Medical and Dental University, Tokyo, Japan; ^2^National Center for Cognitive Behavior Therapy and Research, National Center of Neurology and Psychiatry, Tokyo, Japan; ^3^Department of Health Risk Communication, School of Medicine, Fukushima Medical University, Fukushima, Japan; ^4^Graduate School of Clinical Psychology, Niigata Seiryo University, Niigata, Japan

**Keywords:** anxiety, factor structure, measurement invariance, Japanese, factorial validity

## Abstract

The 7-item Generalized Anxiety Disorder Scale (GAD-7) is commonly used to monitor anxiety symptoms. However, its factor structure has been inconsistent among competing models: unidimensional, two-dimensional, or higher order models. Additionally, it is unknown whether the scale has measurement invariance between populations with and without self-reported psychiatric diagnostic status. Participants were Japanese adults with self-reported anxiety disorder (AD; *n* = 479), self-reported AD and major depressive disorder (MDD; *n* = 314), or without self-reported psychiatric diagnostic status (self-reported non-MDD/AD; *n* = 654), who completed this questionnaire on the Internet. Confirmatory factor analyses showed the higher order model had similar fit indices to the unidimensional and two-dimensional factor models. For the higher order model of GAD-7, metric invariance was supported between the self-reported non-MDD/AD and self-reported AD status groups, and scalar invariance was supported between the self-reported AD status and self-reported AD with MDD status groups. Moreover, convergent and discriminant validity were consistent with previous findings in Western cultures. These results suggest that factor loadings are equivalent and the construct has the same meaning between the self-reported non-MDD/AD and self-reported AD status groups, and the total or sub-scale scores were comparable between self-reported AD status and self-reported AD with MDD status groups. The major limitation of this study is that the participants’ diagnoses were self-reported, not confirmed by clinical structured interview. Further studies that incorporate clinical structured interviews are needed.

## Introduction

The 7-item Generalized Anxiety Disorder Scale (GAD-7; Spitzer et al., 2006) was developed to assess generalized anxiety disorder (GAD) in primary care settings, which has been extensively used. Moreover, the GAD-7 is a useful tool to assess anxiety not only in GAD but also among social anxiety disorder (SAD), panic disorder (PD), and post-traumatic stress disorder (PTSD) (Kroenke et al., 2007). Previous studies using Western populations have reported that the GAD-7 has high reliability and validity for assessing anxiety symptoms (e.g., Ruiz et al., 2011). However, there are uncertainties regarding the following aspects of the GAD-7: (1) its factor structure, (2) its measurement invariance, and (3) the cross-cultural validity of GAD-7.

First, the findings regarding the factor structure of the GAD-7 are not consistent. Some previous studies using Western primary care samples showed a unidimensional factor structure (e.g., Löwe et al., 2008), whereas other studies using Western psychiatric samples reported a two-dimensional factor structure (e.g., Kertz et al., 2013). Beard and Björgvinsson (2014) used a heterogeneous psychiatric sample to suggest that the two-dimensional factor includes the cognitive and emotional experience of anxiety (items 1, 2, 3, and 7) and the physical experience of restlessness (items 4, 5, and 6). However, no studies have examined a possible higher order model consisting of seven primary items, two first-order factors (i.e., the cognitive and emotional experience of anxiety and the physical experience of restlessness), and a single second-order factor, which subsumes both the unidimensional and two-dimensional factor models. We hypothesized that the higher order model would explain these mixed findings, as in a previous study (Taku et al., 2008).

Second, though the growing number of epidemiological and clinical studies use the GAD-7, the measurement invariance (Vandenberg and Lance, 2000) of the GAD-7 across self-reported non-clinical and clinical populations has not been demonstrated. Additionally, little is known regarding whether the factor structure of the GAD-7 is same in patients with only anxiety disorders (AD) and those with AD who have comorbid major depressive disorder (MDD). Beard and Björgvinsson (2014) examined the factor structure of the GAD-7 using heterogeneous psychiatric samples (i.e., each AD, MDD, bipolar disorder, and borderline personality disorder) and showed that a two-dimensional factor model provided the best fit for each AD (GAD, PTSD, SAD, and PD). However, given that Kroenke et al. (2007) showed that participants with each type of AD had moderate levels of depressive and somatic symptoms, it is necessary to know whether the GAD-7 has the same factor structure in these various populations.

Third, there are only a few studies that showed a cross-cultural validation (Löwe et al., 2008; García-Campayo et al., 2010; Donker et al., 2011; Sidik et al., 2012), which is one of measurement validations. Although these previous studies have shown good validity of the GAD-7 in Dutch (Donker et al., 2011), Spanish (García-Campayo et al., 2010), German (Löwe et al., 2008), and Malay samples (Sidik et al., 2012), further research is needed to establish the cross-cultural validity of the GAD-7, especially using Asian sample. Therefore, using Japanese sample, the current study examined the cross-cultural validity of the GAD-7 via examining convergent and discriminant validity. Previous studies reported strong associations between the GAD-7 and other similar measures assessing anxiety symptoms (e.g., Spitzer et al., 2006), measures related to depression (e.g., Löwe et al., 2008), worry, well-being (Kertz et al., 2013; Beard and Björgvinsson, 2014), and disability (García-Campayo et al., 2010) to establish convergent validity. In terms of discriminant validity, a few studies examined associations between the GAD-7 and constructs less closely related concepts, for example borderline-personality traits (Beard and Björgvinsson, 2014).

In this study, we aimed to examine (1) the factor structure of the GAD-7 by comparing unidimensional, two-dimensional, and higher order models via confirmatory factor analysis; (2) the measurement invariance across self-reported non-AD/MDD, AD only, and AD with MDD groups, using multi-group confirmatory factor analysis; and (3) the cross-cultural validity of the GAD-7 by using Japanese sample.

## Materials and Methods

### Participants and Procedure

This study was part of a larger web-based survey to examine the emotions and psychopathology of Japanese populations with and without self-reported psychiatric diagnostic status (Ito et al., 2015a,b) Participant in this study were recruited from panelists registered on Macromill Incorporation and were extracted randomly from the panelist pool on the basis of sex, age, and living area. Macromill Incorporation, which is a Japanese large internet marketing research company, has been used in previous studies (e.g., Sawada et al., 2012). The populations with self-reported psychiatric diagnostic status include patients with PD, OCD, SAD, and MDD. The patients’ diagnoses were self-reported by asking the participants whether they were currently diagnosed with a mental disorder assigned by a medical practitioner, and whether they were using medical services for treatment as following; “Are you currently diagnosed as having Panic Disorder and being treated for the problem in a medical setting?” for example. Of the total participants (*N* = 2,830; 1,547 females, 1,283 males; mean age = 42.4 years, *SD* = 10.4, range = 19–79), this study used three populations: 479 with AD (282 females, 197 males; mean age = 41.89 years, *SD* = 10.08, range = 21–75) including 198 with PD, 116 with SAD, 66 with OCD, and 99 with a comorbidity of AD; 314 with AD and MDD (168 females, 146 males; mean age = 41.58 years, *SD* = 8.52, range = 19–63); and 654 without any psychiatric disorder (361 females, 293 males; mean age = 44.09 years, *SD* = 11.85, range = 19–79). This study was approved by the institutional review board at the National Center of Neurology and Psychiatry (approval number: A2013-002). We obtained informed consent from all participants by their selecting the applicable “agree” option in the online form.

### Measurements

#### Japanese Version of the Generalized Anxiety Disorder Scale-7 (GAD-7)

The Japanese version of the GAD-7 assesses the frequency with which the seven symptoms of anxiety occurred over the last two weeks (Muramatsu et al., 2009) by using a scale from 0 (*not at all*) to 3 (*nearly every day*). Higher scores denote more severe symptoms of anxiety.

#### Measurements for Convergent Validity

To examine the convergent validity of the Japanese version of the GAD-7, we used six measurements. To measure anxiety we used the Japanese versions of the Overall Anxiety Severity and Impairment Scale (OASIS; Ito et al., 2015b) and the State-Trait Anxiety Inventory Form (STAI; Hidano et al., 2000). To measure depression we used the Japanese versions of the Patient Health Questionnaire-9 (PHQ-9; Muramatsu et al., 2007), Kessler Psychological Distress Scale (K6; Furukawa et al., 2008), and Center for Epidemiologic Studies Depression Scale (CES-D; Shima et al., 1985). To measure disability we used the Japanese version of the Sheehan Disability Scale (SDISS; Yoshida et al., 2004).

#### Measurement for Discriminant Validity

To examine the discriminant validity of the Japanese version of the GAD-7, we used the suppression subscale (SUP) of the Japanese version of the Emotion Regulation Questionnaire (Yoshizu et al., 2013). A previous study verified the discriminant validity of the Overall Anxiety Severity And Impairment Scale (OASIS; Ito et al., 2015b), which measures behavioral and functional aspects of anxiety, by examining the association between OASIS and the suppression subscale (SUP), which is a subscale of the Emotional Regulation Questionnaire (Yoshizu et al., 2013).

### Statistical Analysis

First, to examine in detail the factor structure of the GAD-7, namely unidimensional, two-dimensional, and bi-factor models, a confirmatory factor analysis of the GAD-7 was conducted using three populations (*n* = 1,447). The fits of the three factor models to the data were compared using the full information maximum likelihood method. In this analysis, we used the following fit indices: chi-square, root mean square error of approximation (RMSEA), Akaike information criterion (AIC), Bayesian information criterion (BIC), comparative fit index (CFI), and standardized root mean square residual (SRMR). We examined goodness-of-fit indices according to the standards used in past research (Kline, 2015): the chi-square test (*χ*^2^) should not be significant, RMSEA should be <0.10 for acceptable fit and <0.06 for good fit, CFI should be ≥0.90 for acceptable fit and >0.95 for good fit, and SRMR should be <0.10 for acceptable fit and <0.08 for good fit.

Second, to examine the measurement invariance across self-reported non-MDD/AD, self-reported AD status, and self-reported AD with MDD status groups, a multi-group confirmatory factor analysis (Gregorich, 2006) was conducted. We conducted the multi-group confirmatory factor analysis between the self-reported non-MDD/AD and self-reported AD status groups, and then conducted the analysis between the self-reported AD status and self-reported AD with MDD status groups. In this analysis, we constructed six increasingly restrictive models: in Model 1, all parameters were free (configural invariance); in Model 2, loadings were invariant (metric invariance); in Model 3, loadings and intercepts were invariant (scalar invariance); in Model 4, loadings, intercepts, and residuals were invariant (error variance invariance); and in Model 5, loadings, intercepts, residuals, and factor means were invariant (factor variance invariance). As in conventional confirmatory factor analysis, we used RMSEA, AIC, BIC, CFI, and SRMR as fit indices and the same standards for acceptable fit. The criterion for adopting the model is the following: a difference of less than 0.01 in the ΔCFI index supports the less parameterized model (Cheung and Rensvold, 2002).

Finally, we examined the convergent and discriminant validity of the J-GAD-7 via the approach of Westen and Rosenthal (2003). Regarding convergent validity, the J-GAD-7 was hypothesized to correlate strongly with other anxiety measures, such as OASIS and STAI, and depression measures, such as PHQ-9, CES-D, and K6 (Spitzer et al., 2006; García-Campayo et al., 2010; Donker et al., 2011; Beard and Björgvinsson, 2014), and correlate moderately with disability measure such as SDISS (Kertz et al., 2013; Beard and Björgvinsson, 2014; Seo et al., 2014). In terms of discriminant validity, J-GAD-7 was hypothesized to correlate weakly with SUP (Ito et al., 2015b). The strength of the correlation is followings by Evans (1996): 0.20–0.39 is “weak,” 0.40–0.59 is “moderate,” and 0.60–0.79 is “strong.” We summarized the fit between the predicted and observed pattern of correlations and evaluated these correlations using effect size statistics (Westen and Rosenthal, 2003). To qualify construct validity, we used two effect size statistics: *r_alerting–CV_* and *r_contrast–CV_*. The first statistic, *r_alerting–CV_*, is the correlation between the pattern of correlations, which is predicted between the validated measurement and the variables associated with that measurement, and the pattern of observed correlations. The second statistic, *r_contrast–CV_*, accounts for median intercorrelations among measures for examining construct validity, sample size, and the degree of correlations between the target measure and measures examining construct validity.

## Results

### Distribution of Measurement Scores

The distribution of the J-GAD-7, OASIS, STAI, J-PHQ-9, K6, CES-D, SDISS, and SUP scores are shown in **Table 1**. The differences between all measurement scores except SUP were significant among the three populations.

**Table 1:** Distribution of measurement scores in each group.

		Self-reported non-MDD/AD (*n* = 654)	Self-reported AD status (*n* = 479)	Self-reported AD with MMD status (*n* = 314)	*η*^2a^
GAD-7	Total	4.54 (5.16)	8.11 (5.91)	12.89 (5.93)	0.25**
	Cognitive	2.85 (3.34)	5.25 (3.70)	7.98 (3.54)	0.24**
	Physical	1.70 (2.11)	2.87 (2.56)	4.91 (2.78)	0.21**
OASIS	Total	4.68 (4.63)	7.91 (5.24)	12.45 (5.07)	0.27**
STAI	Total	48.08 (12.20)	55.43 (11.76)	62.66 (10.20)	0.19**
PHQ-9	Total	6.96 (6.46)	9.74 (6.95)	16.90 (7.09)	0.28**
K6	Total	10.79 (4.68)	13.68 (5.23)	18.12 (4.81)	0.25**
CES-D	Total	15.75 (10.62)	21.73 (12.90)	33.96 (12.83)	0.26**
SDISS	Total	1.84 (2.71)	3.84 (3.42)	6.83 (3.58)	0.27**
ERQ	SUP	15.86 (4.56)	14.81 (5.12)	16.11 (5.50)	0.01

*MDD, major depressive disorder; AD, anxiety disorder; 95% CI, 95% confidence interval; PHQ-9, patient health questionnaire 9; K6, Kessler Psychological Distress Scale; CES-D, Center of Epidemiologic Studies Depression Scale; ERQ, emotion regulation questionnaire; SUP, suppression subscale. ^a^η^2^ is effect size for ANOVA. ^∗∗^p < 0.01.*

### Confirmatory Factor Analysis

The fit indices of the unidimensional, two-dimensional, and higher order models were compared using the entire sample. The fit indices of the higher order model were similar with those of the unidimensional and two-dimensional models (higher order model: *χ*^2^ (12) = 508.39, *p* < 0.001; RMSEA = 0.12, CFI = 0.97, SRMR = 0.03; unidimensional model: *χ*^2^ (14) = 806.38, *p* < 0.001; RMSEA = 0.14, CFI = 0.95, SRMR = 0.04; two-dimensional model: *χ*^2^ (13) = 508.39, *p* < 0.001; RMSEA = 0.12, CFI = 0.97, SRMR = 0.03). The higher order model, which is composed of two first-order factors (the cognitive and emotional experience of anxiety, physical experience of restlessness) and a single second-order factor, was selected for subsequent analyses because it is suitable for practical use by using both subscale scores and total score.

### Multi-Group Confirmatory Factor Analysis

First, we conducted a multi-group confirmatory factor analysis of the higher order model for the self-reported non-AD/MDD and self-reported AD status groups (**Table 2**). The best-fitting model was Model 2 (metric invariance), wherein the invariant numbers of factors, invariant correspondences of observational variables to latent factors, and invariant factor loadings for all observational variables to loaded latent factors. That is, the factor structures, pattern of loadings, and magnitudes of factor loadings were equivalent between groups.

**Table 2:** Summary of goodness of fit statistics for tested models in multi group analyses.

	*χ*^2^	*P*	df	Δ*χ*^2^	Δdf	RMSEA	AIC	BIC	CFI	SRMR
Self-reported non-MDD/AD group vs. Self-reported AD status group										
Model 1	221.60	<0.001	24			0.121	16537.72	16769.22	0.963	0.039
Model 2	238.99	<0.001	33	17.39	9	0.105	16537.12	16723.32	0.962	0.082
Model 3	299.32	<0.001	37	60.33	4	0.112	16589.44	16755.52	0.951	0.087
Model 4	499.37	<0.001	44	200.05	7	0.135	16775.50	16906.34	0.915	0.115
Model 5	620.80	<0.001	47	121.43	3	0.147	16890.92	16890.92	0.893	0.185
Self-reported AD status group vs. Self-reported AD with MDD status group										
Model 1	241.91	<0.001	24			0.151	12923.93	13139.02	0.940	0.050
Model 2	268.56	<0.001	33	26.65	9	0.134	12932.59	13105.60	0.935	0.066
Model 3	286.12	<0.001	37	17.56	4	0.130	12942.15	13096.45	0.931	0.067
Model 4	334.96	<0.001	44	48.84	7	0.129	12976.99	13098.56	0.919	0.077
Model 5	451.00	<0.001	47	116.04	3	0.147	13087.03	13194.57	0.888	0.185

*MDD, major depressive disorder; AD, anxiety disorder; RMSEA, root mean square error of approximation; AIC, Akaike information criterion; BIC, Bayesian information criterion; CFI, comparative fit index; SRMR, standardized root-mean-square residual; Model 1, all parameters free; Model 2, loadings invariant; Model 3, loadings and intercepts invariant; Model 4, loadings, intercepts, and residuals invariant; Model 5, loadings, intercepts, residuals, and factor means invariant.*

Second, we conducted the multi-group confirmatory factor analysis using only the self-reported AD status and self-reported AD with MDD status groups (**Table 2**). The best fitting model was Model 3 (scalar invariance), wherein the loadings and intercepts were invariant. That is, the factor structures, pattern of loadings, magnitudes of factor loadings, and means of each observed variable were equivalent between groups.

### Convergent and Discriminant Validity

**Table 3** shows the results of convergent and discriminant validity of the Japanese version of the GAD-7. In terms of the construct validity effect size *r_alerting–CV_*, large effect size was found for the GAD-7 (*r_alerting–CV_* = 0.961, *r_contrast_–cv* = 0.912 [95% CI = 0.905, 0.918], *p* < 0.001). Specifically, GAD-7 correlated strongly with OASIS (*r* = 0.81), STAI (*r* = 0.74), PHQ-9 (*r* = 0.85), CES-D (*r* = 0.79), K6 (*r* = 0.80), and SDISS scores (*r* = 0.75), and did not correlated with SUP scores (*r* = −0.04). In the correlational analysis of the cognitive and emotional experience of anxiety and physical experience of restlessness factors, the correlations of both factor scores with measurements of convergent and discriminant validity were similar to those of the Japanese version of the GAD-7 total score (**Table 3**).

**Table 3:** Summary of convergent and discriminant validity analyses.

	GAD-7 Total	F1	F2	Predicted *r*	Raw *λ*	Raw *λ* as integers
OASIS	0.81	0.71	0.64	0.75	0.15	2
STAI	0.74	0.75	0.65	0.75	0.15	2
PHQ-9	0.80	0.83	0.80	0.65	0.05	1
CES-D	0.79	0.73	0.70	0.65	0.05	1
K6	0.81	0.80	0.74	0.65	0.05	1
SDISS	0.75	0.66	0.64	0.65	0.05	1
SUP	−0.04	−0.04	−0.05	0.10	−0.5	−5

*MDD, major depressive disorder; AD, anxiety disorder; 95% CI, 95% confidence interval; PHQ-9, patient health questionnaire 9; K6, Kessler Psychological Distress Scale; CES-D, Center of Epidemiologic Studies Depression Scale; ERQ, emotion regulation questionnaire; SUP, suppression subscale. F1, cognitive and emotional experience of anxiety. F2, physical experience of restlessness.*

## Discussion

This is the first study to show that the fits of the unidimensional, two-dimensional, and higher order models were almost identical among both populations with and without self-reported psychiatric diagnostic status. For higher order models, the factor structure, which means number of factors and correspondent observational variables, was invariant between self-reported non-MDD/AD, self-reported AD status, and self-reported AD with MDD status groups. Additionally, factor loadings and intercepts were invariant between self-reported AD status and self-reported AD with MDD status groups. Moreover, the cross-cultural validity of the GAD-7 was shown using Japanese sample.

The results of factor analysis allow us to use both one factor and two factors scores of the GAD-7. As we examined the higher order model in this study, mixed results about the factor structure of the GAD-7 in previous studies might be resolved. Using one factor score of the GAD-7, we can use a single cutoff point as a criterion, which is shown in previous studies (Spitzer et al., 2006; Kroenke et al., 2007; García-Campayo et al., 2010; Donker et al., 2011; Delgadillo et al., 2012; Kertz et al., 2013). Using two factor scores allows for a more detailed delineation of symptoms. Thus, it is better to use the higher order model for the GAD-7 because we can use it as the unidimensional and two-dimensional factor models.

The results of the measurement invariance testing revealed metric invariance when comparing the GAD-7 scores between self-reported non-MDD/AD and self-reported AD status groups, which indicates that we can expect the same relationships between the construct and the participants responses to the items between these two populations. In comparing the GAD-7 scores between self-reported AD status and self-reported AD with MDD status groups, we found scalar invariance, indicating that we can compare the latent mean of the GAD-7 between these two populations. To date, the GAD-7 has been used within heterogeneous psychiatric samples or non-clinical samples (e.g., Beard and Björgvinsson, 2014). Moreover, the GAD-7 has been used increasingly often in specific subgroups, such as pregnant women (Zhong et al., 2015) and elderly people (Wild et al., 2014). Therefore, the measurement-invariance results indicate that GAD-7 scores can be compared between each population. The analysis of measurement invariance is important because of the aforementioned increasing use of the GAD-7 with various samples.

Finally, we found the cross-cultural validity of the GAD-7 using Japanese sample via examining the convergent and discriminant validity. The findings about the cross-cultural validity of the GAD-7 were added by the current study. Although the construct validity effect size *r_alerting–CV_*, In Japan, no study has previously examined the convergent and discriminant validity of the J-GAD-7. In future studies of the Japanese population, the J-GAD-7 may be used as a valid assessment of anxiety symptoms.

There are several limitations to this study. First, the diagnoses of participants were not assessed by interview. We cannot be certain that the participants truly had AD/MDD because the participants reported their own diagnoses. Internet surveys should therefore incorporate a screening questionnaire that is designed to validate AD and MDD diagnoses (Benson et al., 2009; Miwa, 2012) and query whether the respondent is visiting a psychiatric hospital, in addition to self-reported diagnostic status. Furthermore, the results of this study must be confirmed in populations whose AD/MDD diagnosis is by psychiatric interviews. Second, the self-reported AD status group did not include participants with GAD. Previous studies have suggested that the GAD-7 is a useful tool to assess not only GAD but also SAD, PD, and PTSD (Kroenke et al., 2007). Moreover, Beard and Björgvinsson (2014) reported good sensitivity of the GAD-7 for all patients with GAD, SAD, PD, and PTSD. However, further study is needed to confirm the results of this study using populations that include those with GAD.

## Author Contributions

MI and YT designed the study. MI managed administration of the study, including the ethical review process. KM developed the Japanese version of the 7-item Generalized Anxiety Disorder Scale (GAD-7) used for this study. SD and YT analyzed data. SD drafted the manuscript. MI and YT provided critical comments on the manuscript related to intellectual content. All authors have read and approved the final manuscript.

## Conflict of Interest Statement

The authors declare that the research was conducted in the absence of any commercial or financial relationships that could be construed as a potential conflict of interest.
